# mirMark: a site-level and UTR-level classifier for miRNA target prediction

**DOI:** 10.1186/s13059-014-0500-5

**Published:** 2014-10-25

**Authors:** Mark Menor, Travers Ching, Xun Zhu, David Garmire, Lana X Garmire

**Affiliations:** Department of Information and Computer Sciences, University of Hawaii at Manoa, Honolulu, HI 96822 USA; Molecular Biosciences and Bioengineering Graduate Program, University of Hawaii at Manoa, Honolulu, HI 96822 USA; Epidemiology Program, University of Hawaii Cancer Center, Honolulu, HI 96813 USA; Department of Electrical Engineering, University of Hawaii at Manoa, Honolulu, HI 96822 USA

## Abstract

MiRNAs play important roles in many diseases including cancers. However computational prediction of miRNA target genes is challenging and the accuracies of existing methods remain poor. We report mirMark, a new machine learning-based method of miRNA target prediction at the site and UTR levels. This method uses experimentally verified miRNA targets from miRecords and mirTarBase as training sets and considers over 700 features. By combining Correlation-based Feature Selection with a variety of statistical or machine learning methods for the site- and UTR-level classifiers, mirMark significantly improves the overall predictive performance compared to existing publicly available methods. MirMark is available from https://github.com/lanagarmire/MirMark.

**Electronic supplementary material:**

The online version of this article (doi:10.1186/s13059-014-0500-5) contains supplementary material, which is available to authorized users.

## Background

MicroRNA (miRNA or miR) is one type of non-coding RNA (ncRNA) that regulates gene expression post-transcriptionally [1]. In mammals, the mature form of miRNA is about 22 nucleotide (nt) long and it forms the miRNA-induced silencing complex (miRISC) in combination with argonaute proteins. Using the miRNA sequence as a guide, this miRISC binds to messenger RNAs (mRNAs) to degrade targeted mRNAs or inhibit translation from mRNAs to proteins [2]. There have been over 1,000 annotated miRNAs in humans, and due to the potential to target multiple mRNAs by each miRNA, it is speculated that as much as 60% of mammalian genes are affected by miRNAs [3,4]. Thus abnormal changes in miRNA expression can cause dysregulation of important biological pathways, and are involved in many diseases such as cancers and cardiovascular disease [5-7]. Therefore determination of the target mRNAs of the variety of miRNAs will help understand the development of these diseases.

In mammals, the binding of the miRNA to the mRNA is not perfectly complementary and the underlying mechanism is not fully understood [8]. This makes it a difficult task for computational prediction of the mRNA targets of a particular miRNA. Due to the small number of experimentally verified miRNA-mRNA pairs, early miRNA target prediction methods are rule-based expert systems, such as MiRanda [9].

Currently, a variety of tools have been proposed for miRNA target prediction, based on different methodologies. Among them, TargetScan is a popular method that removes the free energy component and looks for conservation of the 8mer and 7mer seed region (as opposed to conserved miRs in the original version) [3]. TargetScan uses the context scores to rank the predicted targets, based on linear regression trained on microarray data that consider 3′ compensatory pairing (13th to 16th nt), local AU composition, and position effects (distance to closest end of 3′ UTR) [10]. As an improvement, the revised context + score adds predicted seed-pairing stability and target-site abundance [11]. On the other hand, RNAhybrid and PITA are based on thermodynamics. RNAhybrid computes scores based on secondary structure [12], whereas PITA assesses the accessibility of the site (seed match) by the difference between the minimum free energy (MFE) of the duplex and the energy required to unpair and open the target site [13]. Additionally, some recent methods such as mimiRNA [14] and TarBase [15], intend to discover miRNA and mRNA correlations by incorporating large amounts of experimental data. As the number of experimentally verified pairs has increased significantly over the years, statistical or machine learning methods that are data-driven are becoming popular in the area of non-coding RNA classification, including miRNA target prediction [16,17]. An example of the data-driven miRNA target predictor is SVMicrO [18] that combines a larger variety of features than those of rule-based systems with the popular Support Vector Machine (SVM) learning algorithm [19].

A miRNA can potentially bind to multiple sites in the targeted mRNA. Depending on the resolution, one can perceive miRNA target prediction at two levels. At the gene level, one can predict if a given miRNA will target a particular mRNA. At the finer level, we can predict the sites along the interested region of mRNA which a miRNA will interact with. Correspondingly, miRNA target prediction with the classification approach includes at least two types of classifiers: a site-level classifier that predicts the possible target sites along the mRNA, and a gene-level classifier that predicts potential target mRNA overall. For example, DIANA-microT [20] scores individual target sites of a miRNA along the mRNA 3′ UTR and then computes a combined score for the miRNA-mRNA pair overall using an artificial neural network. The distinction between the two levels is important, since the availability of data for training and testing is very different: many experiments identify miRNA-mRNA pairs, but lack results in the locations of the target sites. As a result, the performance of classifiers in relevance to others needs to be evaluated differently.

Most of the experimentally verified target sites remain historically biased toward the 3′ UTRs, although there are growing observations of target sites within coding sequences (CDSs). Therefore in this paper, only target sites in the 3′ UTR will be considered, due to the abundance of training data in 3′ UTR. UTR-level classification will be used in lieu of gene-level classification in this report. However, the method proposed in this study is adaptable to target site prediction within CDSs, when sufficient amount of training data in CDS are available [21].

Despite the considerable advances in miRNA target prediction, there is much room for improving the predictive performance of existing methods [22]. This study aims to improve the predictive performance for miRNA target prediction at both the site and UTR level by considering an extensive list of over 700 predictive features and using the latest collection of experimentally verified miRNA target data. Feature selection is used to find the most relevant, yet least redundant, set of features for site- and UTR-level prediction. Several statistical or machine learning methods are used to integrate the selected features and their performances are compared. Finally, the resulting classifiers called mirMark are compared to existing publically available miRNA target prediction methods. mirMark is demonstrated to have significantly improved predictive performance at both the site and UTR levels.

## Methods

### Data

#### Positive data

The positive data are obtained from miRecords [23] and miRTarBase [24]. At the site-level, only human miRNA-mRNA pairs with validated target site information are taken from miRecords. Since miRecords uses a mixture of older (pre-2011) and current mature miRNA nomenclature, the mature miRNA names are resolved using BLAST: the miRNA sequences given in miRecords are compared with the mature human miRNA sequences obtained from mirBase (v19), the most recent version available during the preparation of this manuscript [25]. Similarly, the target region positions on the 3′ UTR are inferred using BLAST: the target region sequences given in miRecords are compared to the target 3′ UTR sequences obtained from UCSC Genome Browser. Any site-level records with unresolvable miRNA names or target region positions are omitted. The resulting list of 507 miRNA-target site pairs is used as the site-level positive set. This list is provided in Additional file 1: Table S1.

At the UTR level, experimentally validated human miRNA-gene pairs are combined from two sources: (1) all human gene and miRNA pairs from miRecords; and (2) the subset of miRNA-gene pairs that have strong experimental evidence (that is, those that are not labelled as weakly supported) from mirTarBase. In miRecords, again miRNA names that cannot be resolved by comparison with mirBase v16 (version prior to the change of nomenclature) or v19 are omitted, due to the mixture of nomenclature used. In miRTarBase, genes that have multiple distinct/overlapping UTR sequence are omitted, and longest UTRs are used to represent the genes that have some RefSeq UTRs contained within a longer UTR of the same gene. The resulting list of 2,937 miRNA-gene pairs are used as the UTR-level positive set. This list is provided in Additional file 2: Table S2.

#### Negative data

The negative data are generated using mock miRNAs in a manner similar to the approaches used in [9,26]. A mock miRNA is a random permutation of a real mature miRNA sequence that does not have any overlap with the seed sequences from known miRNAs. For each mature miRNA, we use the Fisher-Yates shuffle algorithm [27] to generate random permutations until we find a mock miRNA such that no 7mer in the seed region of the mock miRNA matches a 7mer of the seed region of any real mature miRNA listed in mirBase v19.

At the site level, mock miRNAs are generated for each real miRNA in the site-level positive dataset. For each real miRNA-gene pair in the positive dataset, a corresponding mock miRNA-gene pair is generated and replaces the positive miRNA in the miRNA-gene pair. Negative target regions are then generated for each mock miRNA-gene pair using MiRanda’s alignment algorithm with a minimum alignment score of 155. Doing so allows us to find well aligned target sites and create a balanced set of positive and negative data.

At the UTR level, mock miRNA-gene pairs are generated for each real miRNA-gene pair in the UTR-level positive dataset. The mock miRNAs are generated by randomly permutations of the corresponding real miRNA sequences, as in the site-level negative set. Features in site-level are computed for the UTR level as well, and summary features on these sites are calculated for each pair, with additions of other UTR-level specific features (see the ‘UTR-level features’ section below).

### Site-level features

One hundred and fifty-one site-level features are considered and the full list is given in Additional file 3: Table S3. Below are the descriptions of the site-level features by category.

#### Energy

The total minimum free energy (Duplex_MFE) is computed using RNAduplex [28] on the mature miRNA and the candidate target site (CTS). Region specific minimum free energies are computed by using RNAduplex on the miRNA seed (Seed_MFE) or miRNA 3′ region (3p_MFE) on the CTS. The local minimum free energy of the CTS (Local_target_MFE) is computed by RNAfold [28] on the 100 nt window surrounding the CTS. The local minimum free energy of the CTS whose bases are constrained to be unpaired (Local_cons_target_MFE) is also computed using RNAfold on the 100 nt window surround the CTS. The local opening energy of the CTS (Local_open_energy), a measure of CTS accessibility done in software PITA [13], is computed as the difference between Local_target_MFE and Local_cons_target_MFE.

#### Seed match type

Binary variables specifying the types of seed match in a CTS are computed using MiRanda’s predicted alignment. The types of seed match considered are as follows:

- Seed_match_8mer: p1-p8 Watson-Crick (WC) match
- Seed_match_8merA1: p1 match/mismatch to A, p2-p8 WC match
- Seed_mach7mer1: p1-p7 WC match
- Seed_match7mer2: p2-p8 WC match
- Seed_match7merA1: p1 match/mismatch to A, p2-p7 WC match
- Seed_match6mer1: p1-p6 WC match
- Seed_match6mer2: p2-p7 WC match
- Seed_match6mer1GU: p1-p6 WC match allowing only one GU wobble
- Seed_match6mer2GU: p2-p7 WC match allowing only one GU wobble

#### miRNA pairing

Information of the type of target duplex pairing for the first 20 nt of the miRNA (miR_match_P01 to miR_match_P20) is encoded as an integer-based categorical variable as follows:

- 1: G-C match
- 2: A-U match
- 3: G-U wobble
- 4: mismatch
- 5: gap

Furthermore, the miRNA pairing information is summarized over the seed region, 3′ region, and total miRNA region. This includes the number of G-C matches (Seed_GC), A-U matches, (Seed_AU), GU wobbles (Seed_GU), mismatches (Seed_mismatch), bulges (Seed_bulge), and nucleotides in bulges (Seed_bulge_nt) in the seed region of the miRNA.

#### Target site accessibility

Position-wise and region accessibility values of CTSs are computed using RNAplfold in ViennaRNA package [28] with winsize 80, span 40, and ulength 10. The accessibility of the entire seed region (Seed_acc), the 5′ half of the seed region (Seed_5p_acc), the 3′ half of the seed region (Seed_3p_acc), and position-wise accessibility of each seed position of the CTS (Seed_P01_acc to Seed_P08_acc) are considered. Furthermore, the accessibility of the regions 10 nt upstream (Up_seed_flank_acc) and 10 nt downstream of the seed region (Down_seed_flank_acc), as well as their corresponding position-wise accessibilities (Up_seed_P01_acc to Up_seedP10 and Down_seed_P01_acc to Down_seed_P10) are considered.

#### Target site composition

The nucleotide and dimer composition of the CTS (for example, Target_A_comp, Target_AU_comp), and the flanking 70 nt regions upstream and downstream of the CTS (for example, Up_C_comp, Down_GU_comp) are computed using BioPerl [29]. The flanking AU score described by Grimson *et al.* in [10], which is a weighted count of AU composition flanking the seed region, is also considered.

#### Target site conservation

Per base conservation scores of the human 3′ UTRs are taken from PhastCons46way [30]. The average per base conservation score of the CTS’ seed region (Seed_cons_score), the entire CTS (Target_cons_score), and the 70 nt upstream and downstream flanks of the CTS (Flank_cons_score) are considered.

#### Location of target site

The location of the CTS is considered by computing the distance of the CTS to the closest 3′ UTR end point (Dist_to_end). This distance is scaled by dividing by the length of the 3′ UTR.

#### UTR-level features

A total of 624 UTR-level features are considered. These features include summary statistics of site-level features, 3′UTR related information, and CTSs in 3′ UTRs. Below are the descriptions of the UTR-level features by category.

#### Summary of site-level features

Total, minimum, maximum, and mean values of the 151 site-level features of the CTSs of a miRNA-gene pair are computed. Also considered are the total, minimum, maximum, and mean values of the posterior probability from the random forest-based site-level classifier, MiRanda alignment score, and CTS start and end positions.

#### Other UTR-level features

The length of the 3′ UTR (UTR_length) and the number of CTSs for a miRNA-gene pair (number_sites) are considered. The CTS density (site_density) is computed as number_sites/UTR_length, as done in SVMicrO [18]. Finally, another measure of density is computed by counting the maximum number of CTSs that lie within 100 nt of each other (max_100_sites).

### Feature selection

Two feature selection methods are considered: Correlation-based Feature Selection (CFS) [31] and minimum Redundancy Maximum Relevance (mRMR) [32]. Both methods are based on mutual information, a non-linear measure of correlation. Mutual information values are normalized to be between 0 and 1 using the Linfoot’s method [33].

Both CFS and mRMR seek to balance the relevance and redundancy of the features. Relevance is the correlation of a feature to the class (positive or negative miRNA target), as measured using mutual information. For a feature to be selected, it must be relevant to predicting miRNA targets. On the other hand, redundancy is the correlation between two features. Redundancy between selected features is minimized to keep the number of selected features small.

The key difference between CFS and mRMR is that CFS selects an approximately optimal subset of features that balance relevance and redundancy, whereas mRMR only provides a ranking of features and the number of top ranking features to use is left to be determined by other methods, such as cross-validation.

### Software

RNAduplex, RNAfold, and RNAplfold [28] in Vienna RNA package are used for energy and accessibility computations. Nucleotide composition is computed using BioPerl [29]. Weka 3 data mining software [34] is used for CFS, classifier training, and evaluation. The entropy package [35] in R is used to compute mutual information with the recommended method of Freedman and Diaconis [36] to discretize features of continuous variables.

### Data availability

The detailed instruction and open source code of mirMark for miRNA target prediction are available at [37] and [38]. Additionally, we include the site-level positive data in Additional file 1: Tables S1 and negative data in Additional file 4: Table S4, and the UTR-level positive data in Additional file 2: Table S2 and negative data in Additional file 5: Table S5.

We used the PAR-CLIP data from previous studies [39,40] to compare the performance between mirMark and TargetScan. We obtained one set of data from the supplementary material of Hafler *et al.* [39], and the other datasets from Kishore *et al.* [40], which have the following accession IDs and samples:

- GSM714642 RNA_Ago2_CLIP_completeT1_repA
- GSM714643 RNA_Ago2_CLIP_completeT1_repB
- GSM714644 RNA_Ago2_PAR-CLIP_completeT1_repA
- GSM714645 RNA_Ago2_PAR-CLIP_completeT1_repB
- GSM714646 RNA_Ago2_PAR-CLIP_mildMNase_repA
- GSM714647 RNA_Ago2_PAR-CLIP_mildMNase_repB

## Results

### Structure of mirMark

Most of the identified locations of miRNA targets in the miRNA target database miRecords [23] are in the 3′ UTR region of the mRNA due to historical reason. Although there is evidence that miRNA can also target the 5′ UTR and coding regions of the mRNA, the data are sparse and therefore the focus of this work is on the 3′ UTR, in order to be comparable to the majority of target prediction tools.

**Figure 1:**
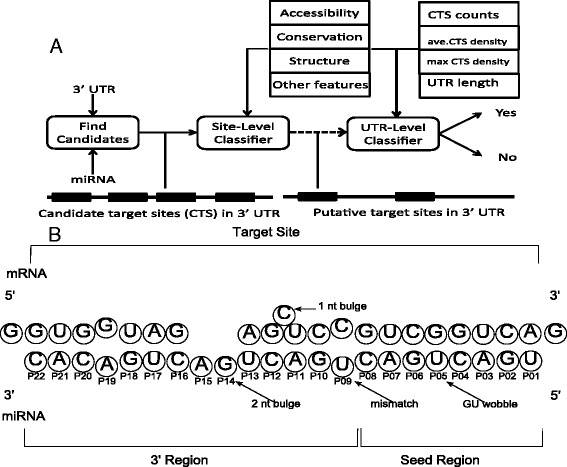
**Structure of mirMark and miRNA-target region duplex. (A)** mirMark consists of two levels of classifiers, site-level and UTR-level, depending on the type of prediction desired. First, candidate target sites (CTSs) of the miRNA on the 3′ UTR are found. The alignment of the CTSs and various other features concerning accessibility, conservation, and structural information are then used by the site-level classifier to find the strongest CTSs. On the other hand, the UTR-level classifier integrates the CTSs to determine if the gene is a target of the given miRNA. **(B)** An illustration of the site-level binding between miRNA and target regions of the 3′ UTR. Information about the type of bindings that occur in the seed region is particularly predictive.

Figure 1A illustrates the structure of miRNA target predictors. First, CTSs of the miRNA on the 3′ UTR of the mRNA are identified. CTSs are found using the alignment algorithm implemented in MiRanda [9]. The alignment favors, but does not require, seed matches to allow for weak seed targets such as 3′ compensatory target sites.

Given the list of CTSs of the miRNA along with their predicted alignments (Figure 1B), the site-level classifier will assign a posterior probability that the given CTS is a target site of the miRNA. This prediction is made on the basis of features such as the presence of a seed match, free energy of the duplex, and the accessibility of the target site.

Finally, given the CTSs and their posterior probability of being a true target as computed by the site-level classifier, the UTR-level classifier will assign a posterior probability that the miRNA targets the mRNA overall. This prediction can be made on the basis of features such as the number of CTSs, the number of CTSs of a particular seed type, and the length of the 3′ UTR. This step allows for the integration of the information provided by the set of CTSs to improve prediction accuracy (Figure 1A).

### Site-level feature selection

Five hundred and seven human miRNA-site pairs are extracted from miRecords [23] along with their experimentally verified duplex structures. Random permutations of the miRNA sequences are used to generate mock miRNAs. To serve as a negative set, 520 mock miRNA-site pairs corresponding to the real miRNA-site pairs are generated using MiRanda’s predicted alignments. The dataset is split in two with 80% for training and cross-validation, and the rest 20% reserved as a hold-out test set for independent evaluation.

**Figure 2:**
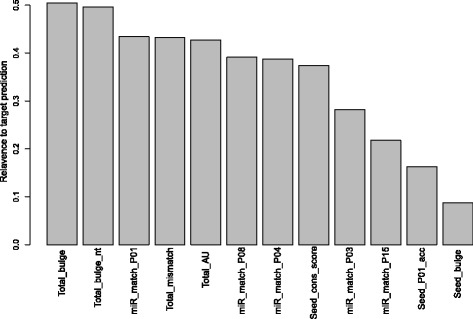
**Selected site-level features.** List of site-level features selected by correlation-based feature selection and sorted by their relevance according to mutual information.

For use in the site-level classifier (Figure 1A), a set of 151 site-level features are generated, which cover a broad spectrum of properties including: energy, seed match, miRNA pairing, miRNA- miRNA-site duplex structure, target site accessibility, and conservation (see details in Methods). Weka’s implementation of CFS [34] is used to select a subset of features that have high relevance to target prediction and yet low redundancy among the other selected features. The 12 selected features with CFS on the training set are listed in Figure 2 along with the selection criterion of their relevance for target prediction. Such relevance is measured by the Linfoot information measure [33] estimated using the entropy package in R [35]. In addition, the mRMR method is used to rank the features and the ranking is provided in Additional file 3: Table S3.

The selected features (Figure 2, Table 1) highlight the importance of the seed region in miRNA targeting, as seven out of the twelve selected features are focused on the seed region. The miR_match_P01, miR_match_P03, miR_match_P04, and miR_match_P08 indicate the types of binding occurring at that particular miRNA position in the miRNA-site duplex, such as a GC match, AU match, or GU wobble. Together with the number of bulges (Seed_bulge), these features are indicative of the stability of the duplex binding in the seed region.

**Table 1:** **Selected site-level features by correlation-based feature selection**

**Feature**	**Description**
miR_match_P01	Match status of miRNA position 1
miR_match_P03	Match status of miRNA position 3
miR_match_P04	Match status of miRNA position 4
miR_match_P08	Match status of miRNA position 8
miR_match_P15	Match status of miRNA position 15
Seed_bulge	Number of bulges in seed region
Total_AU	Number of AU matches in target site
Total_mismatch	Number of mismatches in target site
Total_bulge	Number of bulges in target site
Total_bulge_nt	Number of nucleotides within bulges in target site
Seed_P01_acc	Accessibility score position 1 of seed region
Seed_cons_score	Conservation score of seed region

The two remaining seed-focused features, Seed_cons_score and Seed_P01_acc, are related to conservation and accessibility of the target site (Table 1). Since miRNAs are involved in regulating many vital biological processes, it is not surprising that many target sites are conserved across species and that the average conservation score of the target site’s seed region of the miRNA (Seed_cons_score) is selected. This is in agreement with SVMicrO that also selects the conservation score of the seed region computed using PhastCons28way [18]. The accessibility of the first position of the target site’s seed region (Seed_P01_acc) is selected. Corresponding to our result, it has been shown that the accessibility of the target site’s seed region is highly predictive by analysis of HITS-CLIP data [41].

It is also experimentally observed that a group of target sites exist with relative weak binding in the seed region that is compensated for by strong binding on the target sites overall. Examples are centered and 3′ compensatory sites that have strong pairing on positions 4 to 15 and 12 to 17 of the miRNA, respectively [10,42]. Our CFS selected features provide evidence to support such observations (Figure 2, Table 1). A group of features examine the stability of the target site duplex overall (Total_AU, Total_mismatch, Total_bulge, Total_bulge_nt). Most impressively, a feature for matching at position 15 of the miRNA (miR_match_P15) that is critical to both centered and a 3′ compensatory site is selected by CFS (Figure 2, Table 1).

Compared to the 12 CFS-selected features, the top 12 ranking features selected by another closely related feature selection method mRMR (Additional file 3: Table S3) show 75% agreement. The top 12 features of mRMR do not contain the miR_match_P15, Seed_bulge, Seed_P01_acc, and Total_mismatch features selected by CFS. Instead, mRMR chooses two seed match type features (Seed_match_6mer2GU and Seed_match_7mer2) and two 3′ region features (3p_bulge and 3p_mismatch).

### Evaluation of site-level classifiers

To evaluate different classifiers for site-level target prediction, we perform 10-fold cross-validation on the training set where CFS is performed per fold. We consider four types of linear classifiers: logistic regression (LR) [43], Fisher’s linear discriminant analysis (FLDA) [44], naïve Bayes (NB) [45], and the support vector machine (SVM) [19] with a linear kernel. In addition, we include two non-linear classifiers: the random forest (RF) [46] of 100 random trees and the SVM with the Gaussian radial basis function kernel (Gaussian SVM). Increasing the number of random trees has diminishing returns and it was empirically observed through cross-validation results that 100 random trees are sufficient for site-level classification. The SVM complexity parameter and the Gaussian kernel’s width parameter are selected through cross-validation. The SVM classifiers also include a LR model to approximate posterior probabilities using Platt’s method [47] to improve performance.

**Figure 3:**
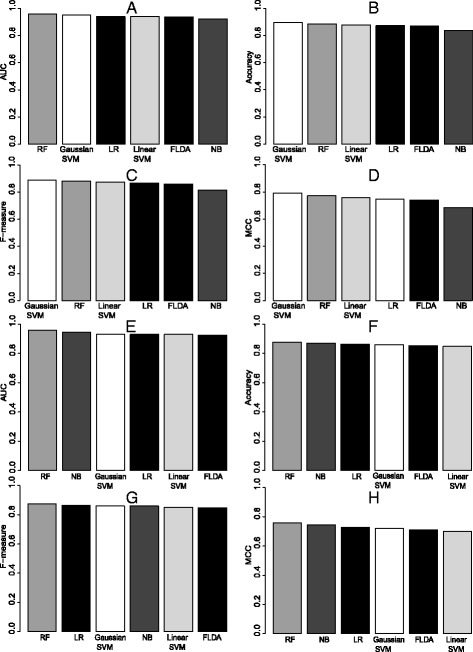
**Evaluation of site-level mirMark.** Performance of site-level mirMark using various classification methods according to **(A)** AUC, **(B)** accuracy, **(C)** F-measure, and **(D)** MCC using 10-fold cross-validation. Similarly, the performance is shown using a hold-out test set **(E-H)**.

Figure 3A to D compare the cross-validation performance of the above six classifiers using four different performance metrics: area under the ROC (AUC), F-measure, accuracy, and Matthew’s correlation coefficient (MCC). The RF classifier performs very closely to the Gaussian SVM in cross-validation. Moreover, the results on the hold-out test set in Figure 3E to H suggest that the RF generalizes better than the Gaussian SVM.

In addition, Gaussian SVM, RF, and LR models are trained using the top ranking mRMR features and the AUC over varying number of features is given in Additional file 6: Figure S1. The AUC achieved is comparable to that of the classifiers using CFS-selected features. Given the practical issue that mRMR only provides a ranking of features and the number of top ranking features to use is left to be determined by other methods (such as cross-validation), we elect to use the features selected by CFS from now on.

### Site-level comparison with other existing methods

Using the site-level independent hold-out test set, we compare the performance of RF, Gaussian SVM, and LR to existing publically available miRNA target prediction software: SVMicrO [18], TargetScan [4], MiRanda [9], RNAhybrid [12], and PITA [13]. Only publically available software is considered in order to obtain predictions on the mock miRNAs. The RF and Gaussian SVM are chosen as they are the top performers in the cross-validation evaluation and LR is chosen as a representative of linear classifiers. Here we only consider site-level predictions in this comparison, and leave UTR-level predictions provided by SVMicrO, TargetScan, MiRanda, and PITA in UTR-level comparisons described later. Note that TargetScan and PITA are seed-level predictors; we extend the seeds to obtain 25 nt long target site regions, so that TargetScan and PITA can be compared with other site-level models.

**Figure 4:**
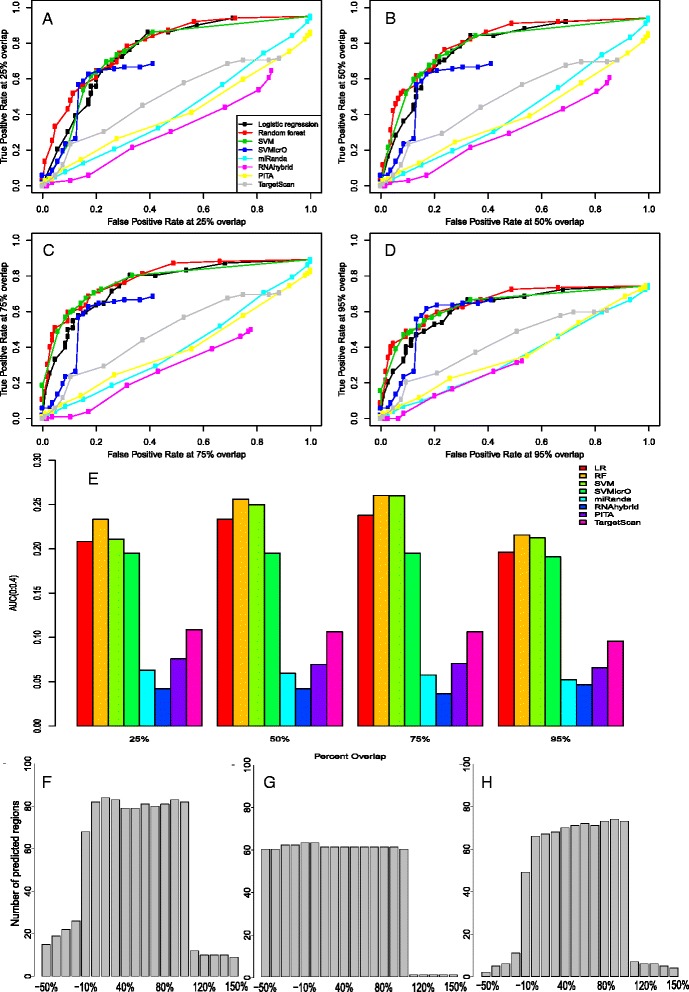
**Comparison to existing methods at site level.** ROC curves for site-level mirMark and existing methods on the hold-out test set using different overlap thresholds to the expected target regions: **(A)** 25%, **(B)** 50%, **(C)** 75%, and **(D)** 95%. **(E)** The AUC of the ROC curves from false positive rate 0 to 0.4 (chosen based on results from a-d) under different overlap thresholds. **(F, G)** The number of predicted regions overlap the expected regions at about 0.6 true positive rate for **(F)** mirMark random forest, **(G)** SVMicro, and **(H)** MiRanda.

The miRNA-site duplexes are predicted, rather than using the experimentally validated duplex structures in miRecords. Therefore the predicted target site locations will not exactly correspond to the expected target site locations in the test set. To allow for this discrepancy, we consider predicted target sites as ‘true target sites’ if they overlap some percentage of an expected target site. Figure 4A to D display ROC curves for minimum overlap thresholds of 25%, 50%, 75%, and 95%. Note that classifiers may not be able to reach a true positive rate of 1.0 due to lack of a predicted target site with sufficient overlap. Since not all methods span the entire false positive rate range, we compute the AUC from the shared false positive rate region from 0.0 to 0.4. The AUC using different minimum overlap thresholds are given in Figure 4E. RF, Gaussian SVM, and LR site-level classifiers clearly outperform the other existing methods in this low false positive rate regime. Among them, the RF classifier achieves the highest AUC across the board.

Next, we investigate the biases that a method may have in the locations overlapping between the expected target sites from miRecords and the predicted target sites. Since the expected target sites vary in length, we divide the length of the expected target sites into 10 equal sized bins. We also include five flanking bins of equal size upstream and downstream of the expected sites from miRecords. The bins count the number of predicted sites that overlap with the coordinates located in the expected target site. We use the predicted target regions given by the methods at about 0.6 true positive rate, according to the 75% overlap ROC curve in Figure 4C. Therefore RNAhybrid is omitted form this analysis. The results are given in Figure 4F to H corresponding to RF, SVMicrO, and MiRanda, respectively. LR and Gaussian SVM have similar results as RF, and they are given in Additional file 7: Figure S2.

In Figure 4F we see that the RF classifier performs very well, as the majority of overlapping predicted target sites are mostly contained within the expected target sites, with only a common overhang at the 5′ end of the expected target site. It also yields most number of predicted regions among the three classifiers tested. In contrast, SVMicrO shows a large overhang at the 5′ end, but a relatively clean cut at the 3′ end that pairs with miRNA seed region (Figure 4G). The clean cut at the 3′ end is expected as potential target sites are first identified by seeking loose seed matches to the miRNA, and then extended by alignment using MiRanda to form a full target region prediction. The results for MiRanda in Figure 4H have relatively similar shape to those for RF in Figure 4F. This is due to the fact that the RF classifier uses MiRanda’s alignment algorithm to identify CTSs. The key difference between MiRanda and the RF classifier is how the CTSs are filtered: MiRanda uses the minimum free energy of the CTS, whereas RF uses a posterior probability estimated from 12 CFS selected-features selected by CFS. This results in a different selection of predicted target sites and therefore some variation in the overlap plots in between the two (Figure 4F, H).

Finally, we also evaluate the performance of mirMark vs. TargetScan using published PAR-CLIP experiment results [39,40]. PAR-CLIP results provide direct ‘finger-print’ information on putative miRNA binding sites genome-wide by pulling down the nucleotide sequences associated with the RNA-binding proteins, making them good additional testing data to detect the sensitivity of the tools. However, note that the direct miRNA and target pairing information is missing in PAR-CLIP data which makes them un-desirable as the positive dataset in mirMark. We randomly selected about 1,000 sites from 100 UTRs detected in PAR-CLIP data as the truth measure, where the UTR sequences are determined by BLAST matching of cross-linked centered regions (CCRs) in PAR-CLIP results. We test the performance of mirMark site-level model on these sites, in comparison with the predictions from TargetScanHuman 6.2 on the same sites. We extend TargetScan predicted seed matching regions to 25 bp, to make it comparable to mirMark. As shown in Additional file 8: Figure S3, in the regions of the high overlap percentages (more than 90%) between prediction and PAR-CLIP results, mirMark with the stringent posterior probability threshold of 0.95 still predicts significantly (27%) more true-positive sites than TargetScan that has a loose threshold of 50% percentile Context + score. This result again shows that mirMark is a better performer at the site level, compared to TargetScan.

### UTR-level feature selection

UTR-level positive training data are taken from miRecords and miRTarBase. These are experimentally validated human miRNA-gene pairs with high confidence. A negative set of mock miRNA-gene pairs associated with the real miRNA-gene pairs is generated by random permutations of the miRNA sequences paired with real UTRs. Like the site-level classification, the dataset is split in two with 80% for training and cross-validation and 20% reserved for independent evaluation.

A total of 624 UTR-level features are considered. They include 3′ UTR level features, such as the density of the predicted targets sites within the 3′ UTR, and summary statistics (maximum, minimum, mean, and summation) based on each of the previously mentioned site-level feature of a miRNA-gene pair (see Methods for details). Also included are the total, minimum, maximum, and mean of the posterior probabilities from the RF site-level classifier on the CTSs of the miRNA-gene pair. The RF site-level classifier is chosen due to its best performance in the site-level evaluation in the previous section.

**Figure 5:**
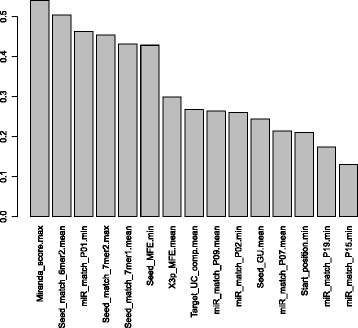
**Selected UTR-level features.** List of UTR-level features selected by correlation-based feature selection and sorted by their relevance according to mutual information.

A total of 15 UTR-level features are selected by performing CFS on the training set (Table 2), and they are ranked in Figure 5 along with their relevance to target prediction. The stability of seed regions of the CTSs is again clearly important among the selected 15 features. Four of the features are either related to seed match or MFE in seed regions. The former category is demonstrated by the proportion of CTSs that have some form of 6mer or 7mer seed match (Seed_match_6mer2.mean and Seed_match_7mer1.mean) and the existence of a CTS with a 7mer match in positions 2 to 8 of the miRNA (Seed_match_7mer2.max). MFE of seed regions is important, demonstrated by the selected features of the minimum MFE (Seed_MFE.min) and mean number of G-U matches (Seed_GU.mean) within the CTSs’ seed regions. These features also give an aggregated account to the overall stability of the CTSs. Lastly, three features for binding that occurs on seed positions of the miRNA (miR_match_P01.min, miR_match_P02.min, and miR_match_P07.mean) are selected, as expected.

**Table 2:** **Selected UTR-level features by correlation-based feature selection**

**Feature**	**Description**
Miranda_score.max	Maximum alignment score between miRNA and target sites
Seed_match_6mer2.mean	Proportion of target sites with P02-P07 WC match
miR_match_P01.min	Match status of miRNA position 1
Seed_match_7mer2.max	Proportion of target sites with P02-P08 WC match
Seed_match_7mer1.mean	Proportion of target sites with P01-P07 WC match
Seed_MFE.min	Minimum MFE of seed region of miRNA:site duplexes
X3p_MFE.mean	Mean MFE of 3′ region of miRNA:site duplexes
Target_UC_comp.mean	UC dimer composition of the CTS
miR_match_P09.mean	Match status of miRNA position 9
miR_match_P02.min	Match status of miRNA position 2
Seed_GU.mean	Mean number of GU matches in target site seed regions
miR_match_P07.mean	Match status of miRNA position 7
Start_position.min	Minimum distance of target sites to the 5′ end of the 3′ UTR
miR_match_P19.min	Match status of miRNA position 19
miR_match_P15.min	Match status of miRNA position 15

As mentioned in the results of site-level feature selection, there exist target sites with relative weak binding in the seed region that are compensated for by strong binding on the target site overall. There are three selected features focused on the 3′ region of the miRNA (Table 2, Figure 5). The mean MFE (X3p_MFE.mean) in the 3′ region of the CTSs is indicative of the stability of the duplex beyond the seed region. Furthermore, the existence of a CTS with good binding on positions 15 and 19 of the miRNA (miR_match_P15.min and miR_match_P19.min) provides strong evidence of the importance of central region and 3′ compensatory pairing respectively, which were observed by others experimentally [10,42]. Additionally, the maximum MiRanda alignment score (Miranda_score.max) of the CTSs provides evidence of the overall presence of stable CTS bindings.

Finally, the literature has shown that the location of a target site is biased toward the ends of the 3′ UTR [4] and CFS has partially detected this bias through its selection of a feature indicating the distance to the 5′ end of the 3′ UTR to the closest CTS (Start_position.min in Table 2).

### UTR-level comparison with existing methods

Using the UTR-level hold-out test set that is independent of mirMark models, we compare the performances of RF, LR, and Gaussian SVM classifiers in mirMark to those of TargetScan, SVMicrO, MiRanda, RNAhybrid, and PITA. With the exception of RNAhybrid and TargetScan, all other methods provide predictions at the UTR level by integrating site-level evidence. RNAhybrid produces UTR-level predictions solely based on the minimum MFE in predicted target site.

**Figure 6:**
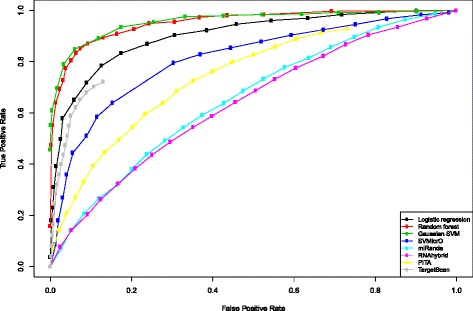
**Comparison to existing methods at UTR level.** ROC curves for UTR-level mirMark and existing methods on the hold-out test set.

The performances of the UTR-level classifiers on hold-out test set are shown by ROC curves in Figure 6. RF, LR, and Gaussian SVM classifiers of mirMark clearly dominate over the other existing methods that are publicly available for comparison, including TargetScan, SVMicrO, MiRanda, RNAhybrid, and PITA. Among these three classifiers of mirMark, the Gaussian SVM classifier achieves the highest AUC of 0.958, closely followed by the AUC of 0,953 from RF, and subsequently 0.901 from LR. For both site-level and UTR-level classifiers, RF and Gaussian SVM classifiers achieve strong performance. However, the Gaussian SVM performs marginally better for the UTR-level classifier.

Given the wide application of TargetScan, we next ask what is the sensitivity of mirMark in the false-negative UTR targets of TargetScan? To answer this question, we obtain 19641 UTR targets detected by PAR-CLIP [39,40], among which 1757 UTR targets are not predicted by TargetScanHuman version 6.2. We randomly select 300 of these targets as inputs to mirMark’s UTR-level classifier, in combination with miRNAs in miRBase. We choose the posterior probability from the best match for each UTR target, and plot the posterior probability density distribution in Additional file 9: Figure S4. Most of the UTR targets from PAR-CLIP are detected with high confidence from mirMark.

## Discussion

### Mock miRNA based vs. real biologically negative dataset

It is debatable what type of negative datasets are the best for miRNA target predictions, with the machine learning approach. True negative target data are simply the complement of the true positive target data, whereas the set of ‘true positive target data’ are an unknown entity. The true negative target dataset is quite large. To obtain a balanced classification design, a subset of negative data must be chosen in an unbiased manner as the negative training data.

We have taken the mock miRNA and real targets pairing approach to generate the negative data, whereas others used negative data with some experimental support, such as using real miRNAs and genes with no experimental evidence of being the targets of miRNAs. In order to investigate which approach applies better in partner with the positive data that we use, we compare mirMark’s negative dataset composed of mock miRNA and real targets pairs, with another approach using real miRNAs and genes lacking experimental evidence of being miRNA targets, similar to Marin *et al.* [41] and Ritchie *et al.* [48]. In this alternative approach, we combine all potential targets from miRecords, miRTarBase, and 7 PAR-CLIP datasets [39,40] to obtain a set of miRNA targets, and then exclude them from RefSeq genes to get the ‘biologically negative targets’. We then randomly pair the real positive miRNAs with these biologically negative targets to generate a biologically negative dataset. This biologically negative dataset is then split into training/testing data, with/without combination with the mirMark’s mock miRNAs as the testing/training data. This results in four scenarios: (1) mock miRNAs for both model building and validation (the mirMark method); (2) biologically negative data for both model building and validation; and (3, 4) two more cases with mixed mock miRNAs and biologically negative data for model training and validation, and vice versa. We show that the mirMark mock miRNA negative data approach has the best predictive performance on testing dataset (Additional file 10: Figure S5) among the four combinations, including the scenario where biologically negative data are used for both model building and testing-data validation. This consolidates the suitability of using mock miRNA real targets pairs as negative data, in partner with the experimentally validated positive data from mirRecords and mirTarBase.

Due to the use of miRanda to align CTS for the site-level negative data, biases might be introduced for features relevant to the miRanda algorithm. To minimize possible biases, we use a stringent miRanda alignment score threshold for the negative data. Only CTS with a miRanda score of 155 or higher are allowed as part of the negative data. Such a stringent threshold potentially dampens or dilutes the importance of seed matching. Even so, the selected features still (Figure 2, Table 1) highlight the importance of the seed region in miRna targeting, as seven out of the twelve selected features are focused on the seed region.

### Relevance of selected features to classes

The correlations of the site- and UTR-level features to the class in Figures 2 and 5, respectively, are not very strong, with values around 0.55 or below. This suggests that an individual feature by itself is not a very strong predictor of miRNA targets. Rather, a set of features is necessary to make target predictions reliably. This is supported by the prediction results given in Figures 4 and 6, where the target predictions based on duplex MFE (MiRanda and RNAhybrid) and accessibility (PITA) underperform the machine learning-based predictors that integrate multiple features (mirMark and SVMicrO).

### Comparison of mirMark classification methods

Random forest and Gaussian SVM are two top classifiers that have very close predictive performances overall. At site level, the non-linear methods of mirMark are only marginally superior in cross-validation (Figure 3), regardless of metrics, suggesting that the decision boundary between target sites and non-target sites is nearly linear. Since the SVM learning algorithm approximately optimizes accuracy, it is not surprising the non-linear Gaussian SVM outperforms all other methods in accuracy. Also the training data are balanced between target site and non-target site examples, this may explain why Gaussian SVM yields better F-measure and MCC than other classifier. It has been shown that Platt’s method is unreliable at estimating posterior probabilities from SVM outputs [49], which may explain the slight underperformance of Gaussian SVM compared to random forest in the AUC measure that relies on the posterior probability estimates. However, there is a drop in SVM performance from cross-validation to test results at site level. This may be due to selection bias in the cross-validation results, which was used to select the SVM parameters. The results on the test set suggest that some overfitting of the SVM models is caused by choosing the SVM model with the best observed AUC in cross-validation. On the other hand, random forest is an ensemble of decision trees where the classification is determined by most popular vote among all model trees. It is known to converge without the overfitting problem [46]. This advantage of random forest is exhibited in the hold-out testing set at site-level.

### Site-level vs. UTR-level predictions of mirMark

None of the posterior probability features, the outputs of the RF site-level classifier, are selected by CFS for UTR-level classification. This suggests that UTR-level target prediction can be largely independently of results out of the site-level target prediction. Indeed, the majority of the CFS-selected UTR-level features are highly correlated to the posterior features, as the heatmap in Additional file 11: Figure S6 shows. Thus we propose that necessary predictive information for the UTR-level is contained in the summary site-level features and the results of a site-level classifier are not an absolute requirement for UTR-level target prediction. In fact, two summary statistics of site-level features (miR_match_P01.min and miR_match_P15.min) are selected as the UTR-level features, confirming the importance of complementary matching in both the seed region and positions 13 to 16 in the central region that were observed by others experimentally [10,42]. The finding that miR_match_P15 is an important feature has prompted us to conduct more detailed analysis on the type of binding between miRNA and targets at this position. As shown in Additional file 12: Figure S7, this position has slightly more match (23% G-C matches and31% A-U matches) than the no-match cases (15% G-U wobbles, 27% mismatches and 3% gaps), supporting the result that miR_match_P15 is an important feature in the model.

### Comparison to SVMicrO

SVMicrO is a recent SVM based miRNA target prediction tool that showed superior performance to earlier methods such as TargetScan and PicTar [18]. Compared to the other programs discussed in this paper, mirMark is algorithmically more similar to SVMicrO. Both methods start with a large variety of features and use feature selection methods to select a smaller subset of features for use in the site- and UTR-level classifiers. mirMark predictors and SVMicrO share a common structure of using MiRanda to identify CTSs and using machine learning methods to train site- and UTR-level classifiers.

Unlike the mock miRNA approach for generating negative data in mirMark, SVMicrO created a negative dataset based on genes that positively correlated to miRNAs in miRNA expression microarray experiments [18]. However, compared to the mock miRNA approach, SVMicrO’s approach may be biased to experimental conditions, as well as too restrictive since there may exist many true negative data that are not positively correlated in microarray experiments. These may also explain our observations that SVMicrO performs better than TargetScan at the site level (Figure 4), but not at the UTR level (Figure 6).

Besides the different datasets from SVMicrO, mirMark has improved method design, which may also lead to the significantly better performance than SVMicro. SVMicrO uses MiRanda to identify potential seed matches. This prevents SVMicrO from identifying target sites that have a weak seed match, such as 3′ compensatory and centered sites [10,42]. Thus one improvement of the mirMark predictors is the use of MiRanda to identify full CTSs in order to find strong binding regions overall but not just in the seed region. Another improvement arises from the much more features considered by mirMark. At the UTR level, the feature selection conducted for SVMicrO only consists of 60 features relating to the total number of predicted target sites of particular seed types, the top score of the sites provided by the site-level classifier, and the density of the predicted sites [18]. Whereas the feature selection conducted for mirMark casts a wider net of 624 features, including summary statistics of every site-level feature considered. This allows the selection of a subset of UTR-level features that are more predictive than those of SVMicrO.

### Potential limitations of mirMark and future work

As mentioned earlier, mirMark is built on the machine learning approach, thus the results of the model are dependent on input data, like all statistical models. Positive data for mirMark are obtained through the combined results from miRecords and a stringent selection of miRTarBase. Unavoidably, the miRNA and target interactions from these databases may simply reflect the miRNAs and genes of interest to the experimentalists who performed the validation [50], and they may not be representative of the landscape of target interactions in general. Furthermore, the choice of the negative dataset potentially introduces bias in the model. mirMark uses the mock miRNA approach for negative dataset generation. Mock miRNAs are *in silico* constructions and are not found in nature, according to current knowledge of miRNAs, it is possible that they have different sequences and properties compared to ‘true negative miRNAs’, which we do not know the complete set yet. We used the mock miRNA to pair with true positive targets in the generation of negative data, thus any potential target bias from the positive dataset is carried over to the negative dataset as well. Additionally, miRanda is used to find candidate target sites with seed matching in both positive and negative datasets of mirMark, therefore the selected features may be biased against seed matching but favor other features that are not used by miRanda filtering. This could explain why *Total_AU*, the number of AU base matches between the miRNA and the target, is selected in our site-level classifier and has better relevance to the classification outcome than other features that are related to seed matching.

While recognizing the potential problems due to the input data, we are optimistic that the machine learning approach is the state-of-art methodology for more accurate miRNA target prediction. The quality and quantity of training data are continuously improving, as more and more miRNA-target interaction data are recorded by databases such as miRecords and miRTarBase. We plan to maintain and update mirMark regularly as new training data become available. Moreover, we will expand mirMark from predicting human to other species, such as mouse, in the near future.

## Conclusions

A new site- and UTR-level miRNA target tool, mirMark, is proposed. It initially considers an extensive list of over 700 features. This list is narrowed down to find the sets of the most relevant and minimally redundant features using feature selection. Evaluation of mirMark at the site and UTR levels reveals the overall superior performance of the random forest classification method. Furthermore, mirMark shows significant improvement in predictive performance compared to existing publically available methods for human miRNA target prediction.

## Additional files

Additional file 1: Table S1. CSV file of site-level human miRNA-target site pairs from miRecords. Additional file 2: Table S2. CSV file of UTR-level human miRNA-gene pairs from miRecords and miRTarBase. Additional file 3: Table S3. Description of 151 site-level features along with their mRMR ranking computed using 10-fold cross-validation on the site-level training set. Additional file 4: Table S4. CSV file of site-level human mock miRNA-target site negative pairs. Additional file 5: Table S5. CSV file of UTR-level human mock miRNA-gene pairs. Additional file 6: Figure S1. AUC of random forest, Gaussian SVM, and logistic regression models using the top ranked mRMR features. Additional file 7: Figure S2. The number of predicted regions overlaps the expected regions at about 0.6 true positive rate for **(a)** mirMark SVM and **(b)** mirMark logistic regression. Additional file 8: Figure S3. mirMark and TargetScan site-level comparison using cross-linked centered regions (CCRs) from 100 UTR targets in PAR-CLIP experiments. Additional file 9: Figure S4. Probability density plot of mirMark UTR level prediction on PAR-CLIP data not detected by TargetScan. Additional file 10: Figure S5. Comparison of predictive performance of models generated from two different types of negative datasets. The mock-miR based negative data are split into training/test sets, with/without biological negative data as test/training sets. Additional file 11: Figure S6. Heatmap of the Linfoot information measure between site-level mirMark random forest posterior probability outputs and the 15 CFS-selected UTR-level features. Additional file 12: Figure S7. Summary of the types of matches in miRNA position 15 (miR_match_P15) from the site-level data.
