# Supplementary figures and images for: mirMark: a site-level and UTR-level classifier for miRNA target prediction
Source: Genome Biol. 2014 Oct 25;15(10):500. doi: 10.1186/s13059-014-0500-5 (PMC4243195; doi:10.1186/s13059-014-0500-5)

Figure S1

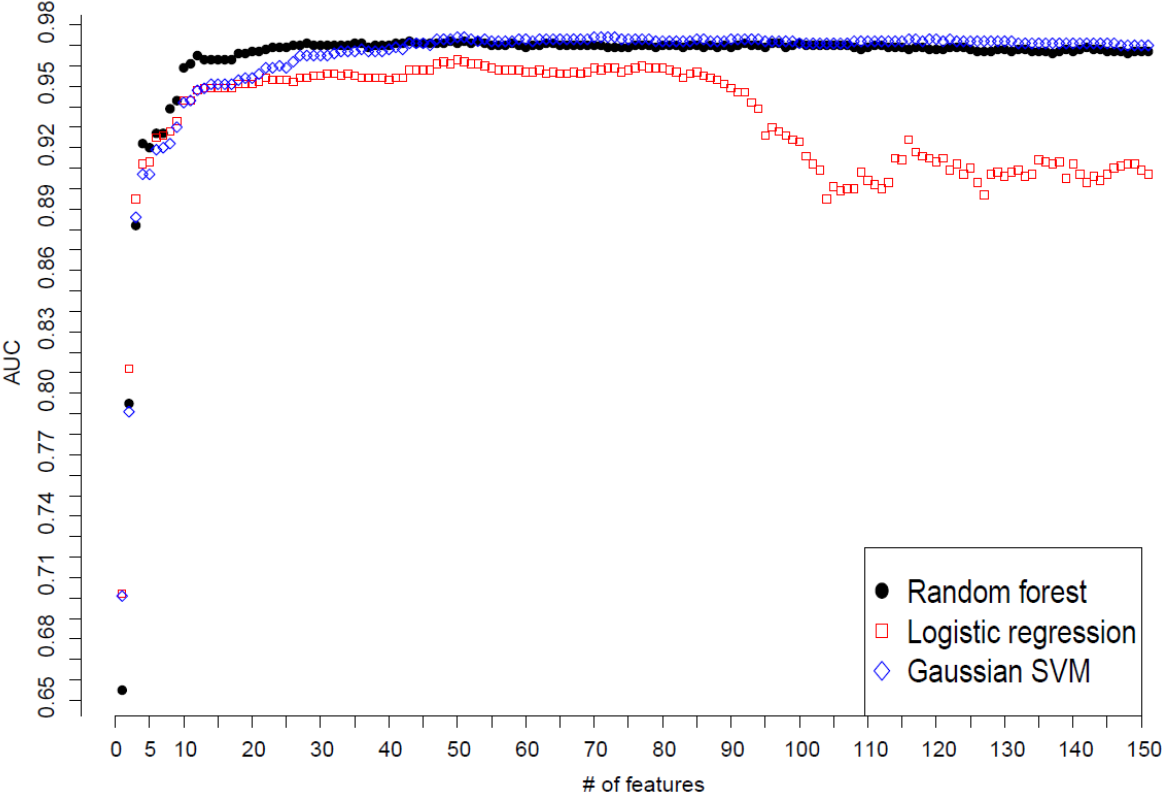

Supplement: Additional file 6: Figure S1. — AUC of random forest, Gaussian SVM, and logistic regression models using the top ranked mRMR features. [file 13059_2014_500_MOESM6_ESM.pdf]

Figure S2

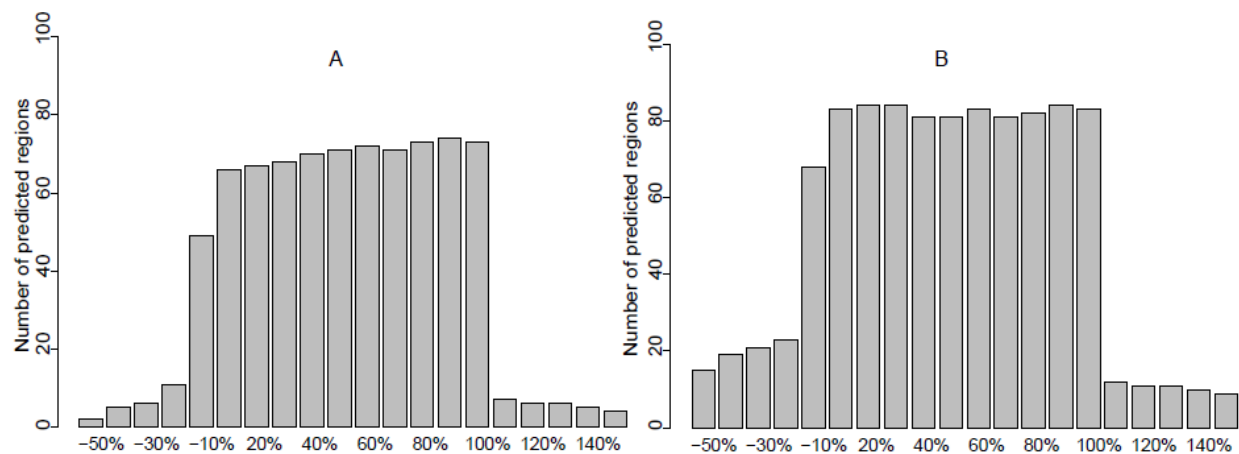

Supplement: Additional file 7: Figure S2. — The number of predicted regions overlaps the expected regions at about 0.6 true positive rate for (a) mirMark SVM and (b) mirMark logistic regression. [file 13059_2014_500_MOESM7_ESM.pdf]

Figure S3

# PAR-CLIP data comparison

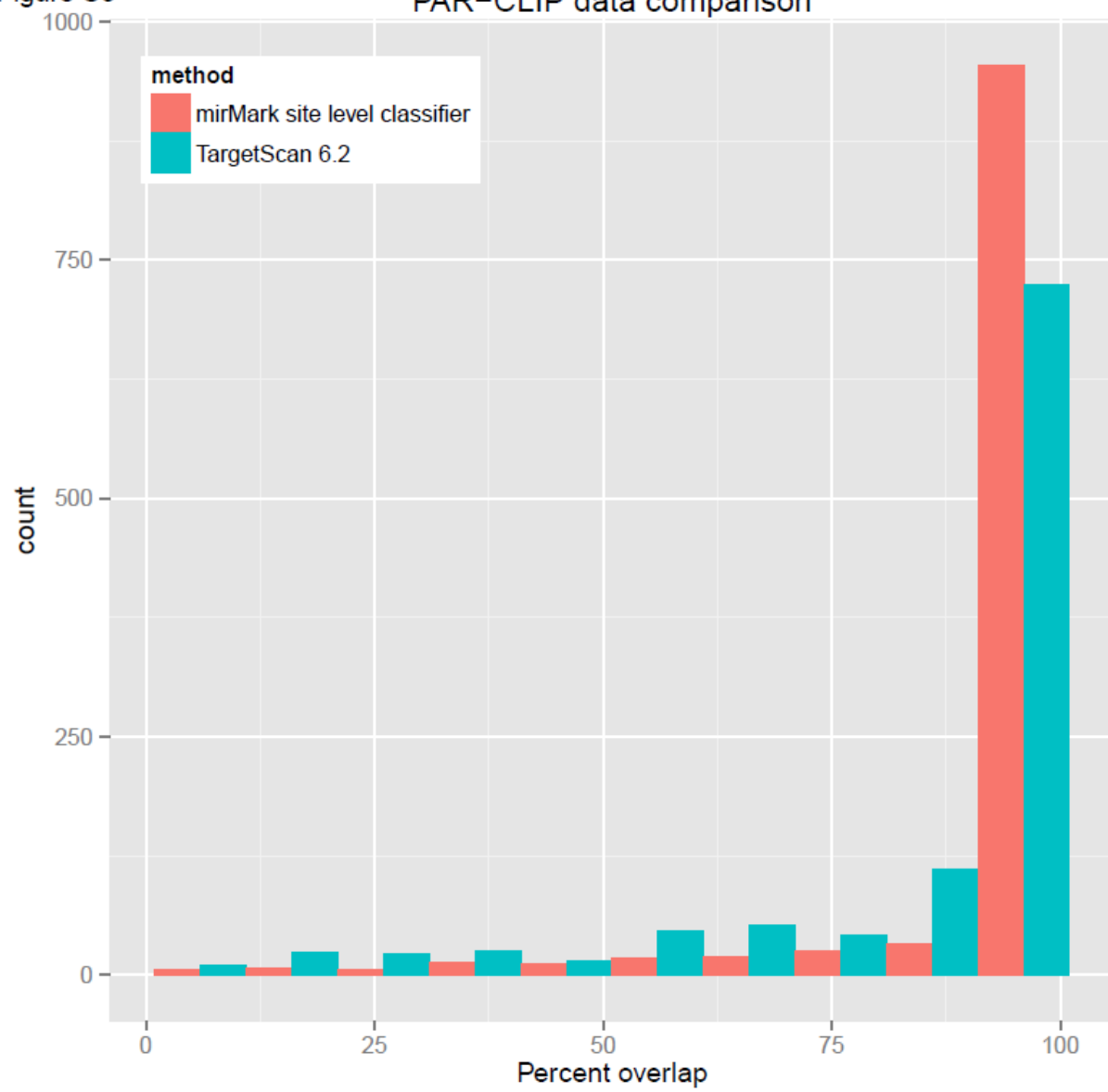

Supplement: Additional file 8: Figure S3. — mirMark and TargetScan site-level comparison using cross-linked centered regions (CCRs) from 100 UTR targets in PAR-CLIP experiments. [file 13059_2014_500_MOESM8_ESM.pdf]

Figure S4

MirMark UTR predictions on  
PAR-CLIP data not found by TargetScan

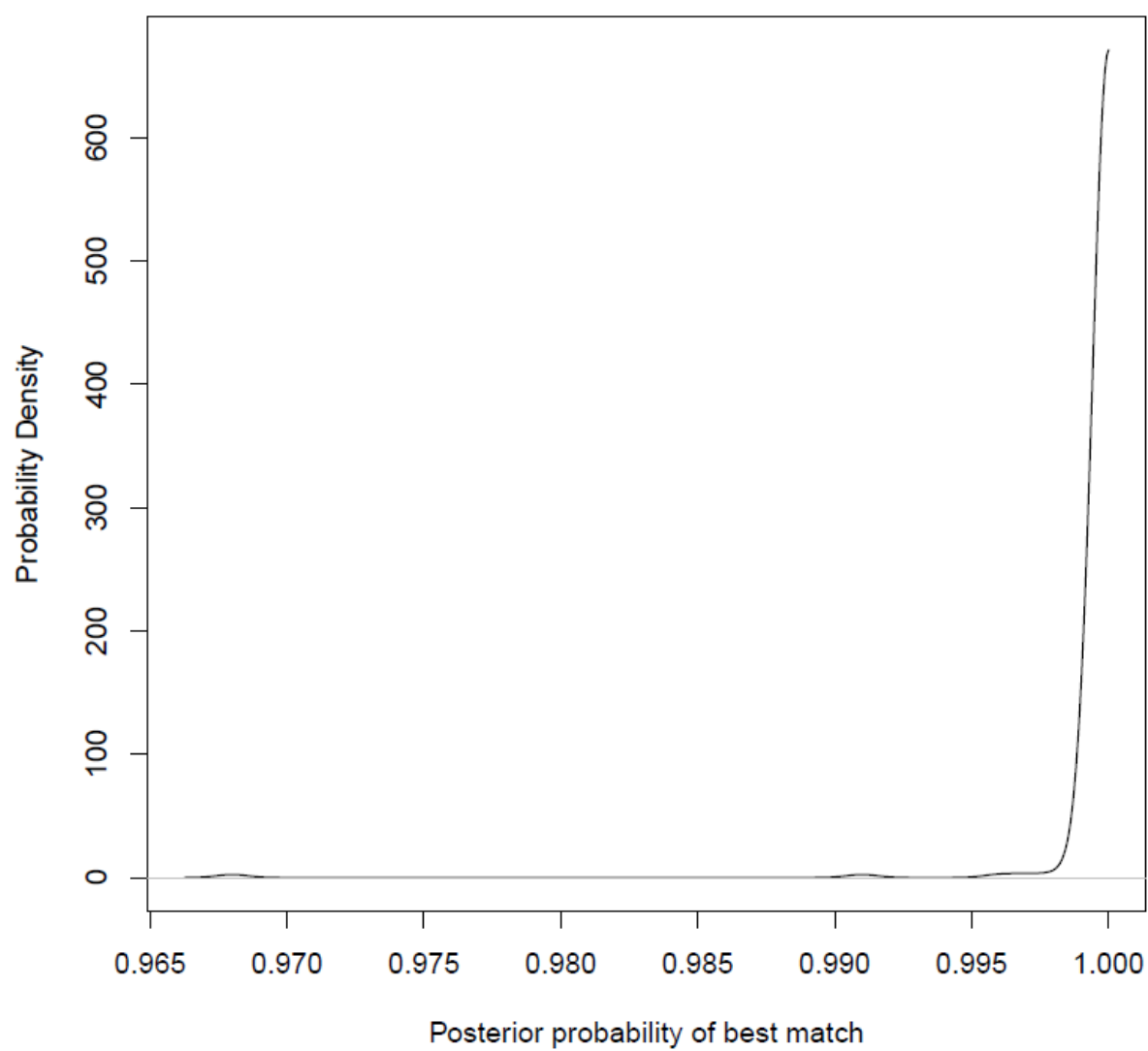

Supplement: Additional file 9: Figure S4. — Probability density plot of mirMark UTR level prediction on PAR-CLIP data not detected by TargetScan. [file 13059_2014_500_MOESM9_ESM.pdf]

Figure S5

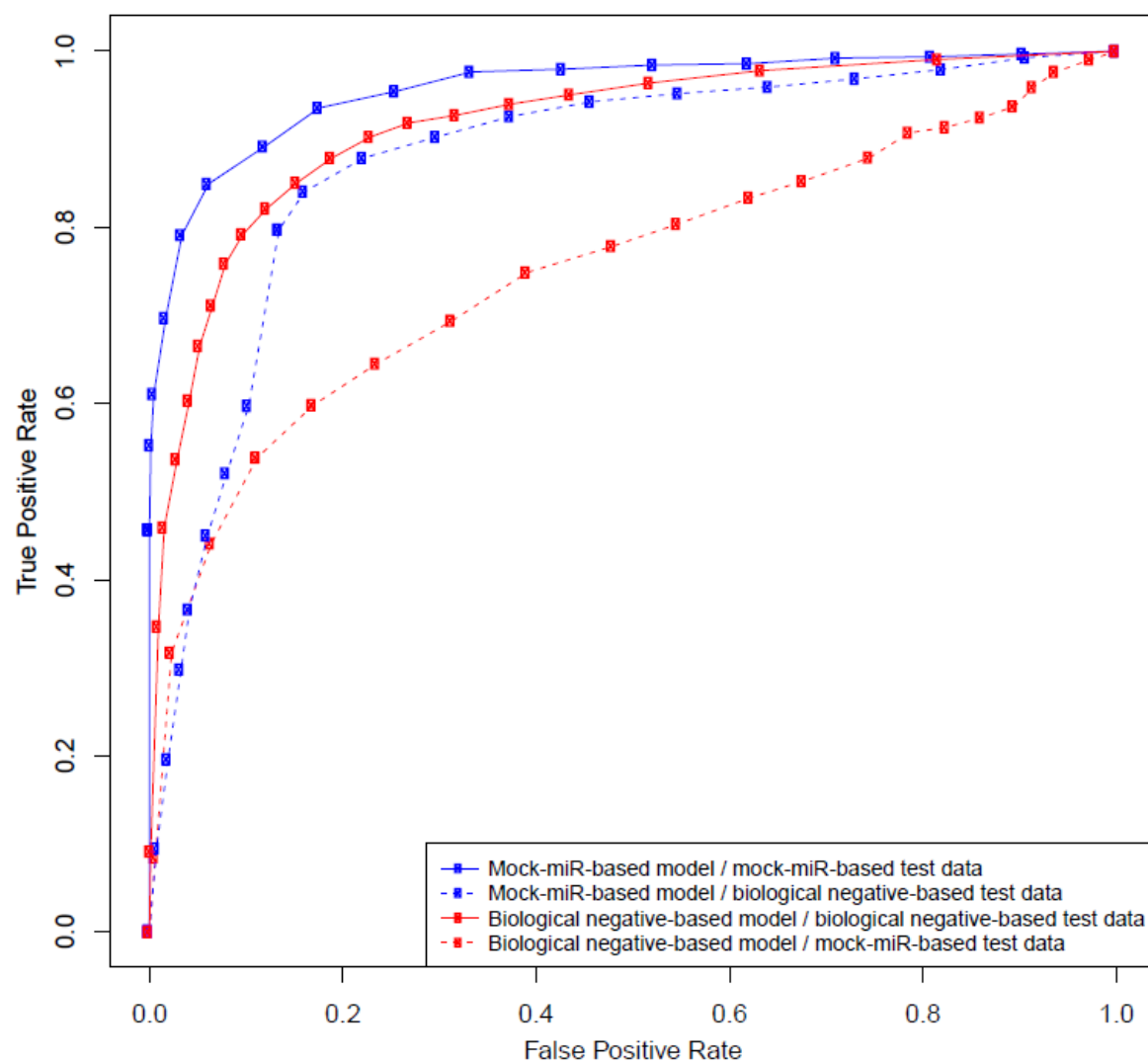

Supplement: Additional file 10: Figure S5. — Comparison of predictive performance of models generated from two different types of negative datasets. The mock-miR based negative data are split into training/test sets, with/without biological negative data as test/training sets. [file 13059_2014_500_MOESM10_ESM.pdf]

Figure S6

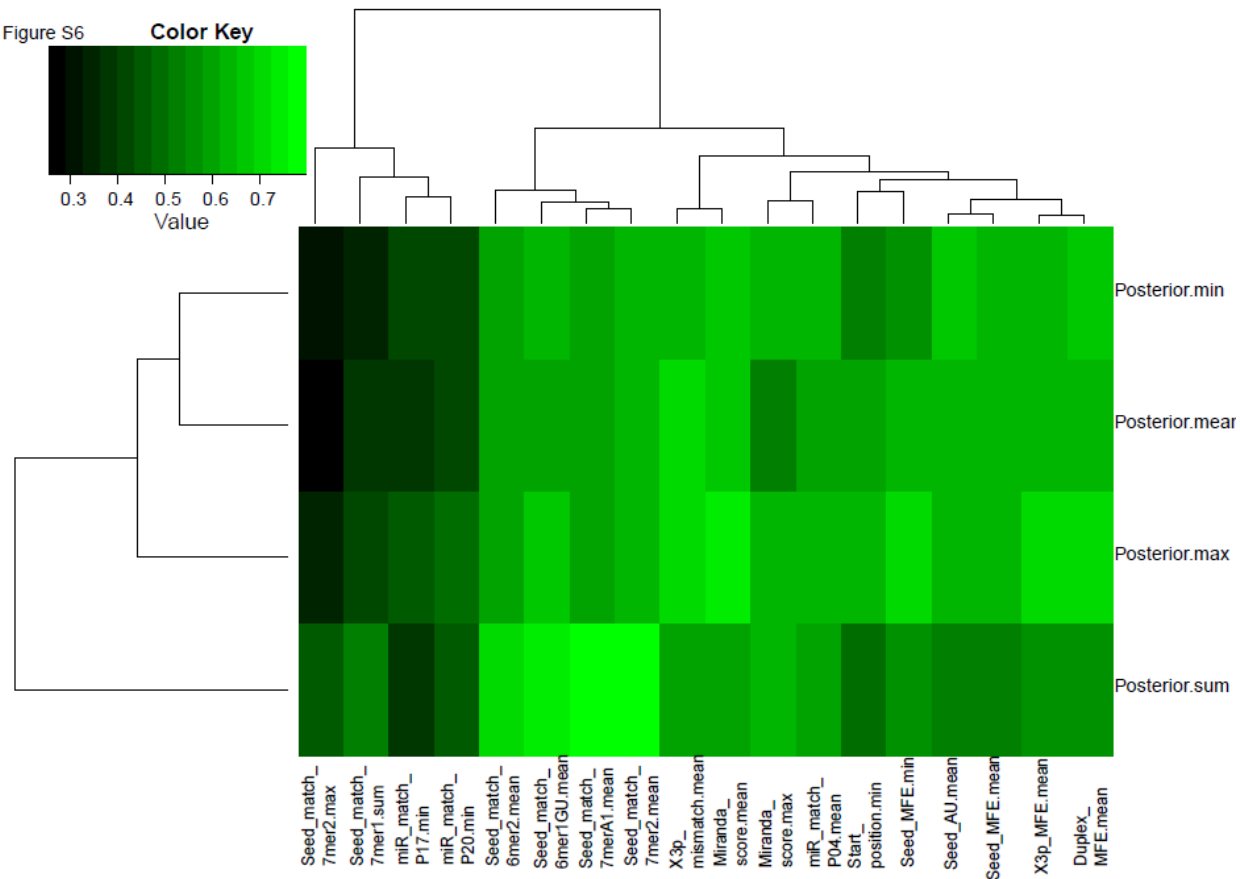

Supplement: Additional file 11: Figure S6. — Heatmap of the Linfoot information measure between site-level mirMark random forest posterior probability outputs and the 15 CFS-selected UTR-level features. [file 13059_2014_500_MOESM11_ESM.pdf]

Figure S7

miR\_match\_P15

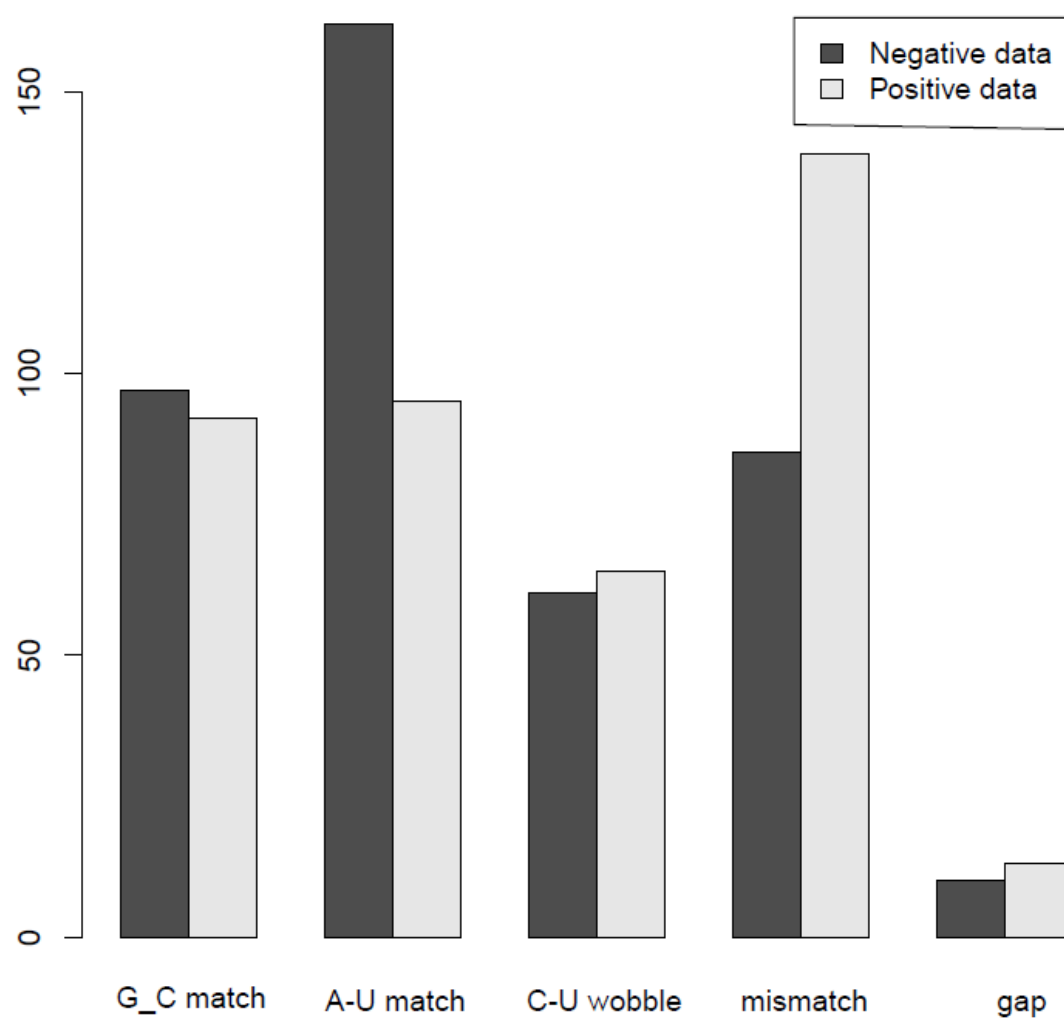

Supplement: Additional file 12: Figure S7. — Summary of the types of matches in miRNA position 15 (miR_match_P15) from the site-level data. [file 13059_2014_500_MOESM12_ESM.pdf]
